# Serum cytokine profiles in children with IgA vasculitis with nephritis

**DOI:** 10.17305/bb.2024.11081

**Published:** 2024-09-03

**Authors:** Yanyan Jin, Xue He, Weiqiang Lin, Zhaoyang Peng, Wei Li, Wenqing Xiang, Zhihui Chen, Haidong Fu, Jianhua Mao

**Affiliations:** 1 Department of Nephrology, The Children’s Hospital, Zhejiang University School of Medicine, National Clinical Research Center for Child Health, Hangzhou, China; 2 International Institutes of Medicine, The Fourth Affiliated Hospital, Zhejiang University School of Medicine, Hangzhou, China; 3 Department of Clinical Laboratory, The Children’s Hospital, Zhejiang University School of Medicine, National Clinical Research Center for Child Health, Hangzhou, China; 4 Department of Infection Management, Wenzhou People’s Hospital, Wenzhou, China

**Keywords:** IgA vasculitis with nephritis (IgAVN), high-throughput cytokine chip, signaling pathway, molecular markers

## Abstract

This study aimed to discover novel serum biomarkers for IgA vasculitis with nephritis (IgAVN). The serum of IgA vasculitis (IgAV) patients without nephritis and IgAVN patients not treated with glucocorticoids was analyzed for 440 proteins using a novel quantitative planar protein microarray. To verify the biomarkers, semiquantitative immunofluorescence analysis was performed on selected differential cytokines in a separate cohort of kidney tissue samples. A total of 41 proteins were differentially expressed between the IgAVN and IgAV groups out of the 440 proteins analyzed. Five differentially abundant proteins, including VEGF R3, ADAM12, TIM-3, IL-12p40, and CEACAM-5, were further validated by semiquantitative immunofluorescence analysis in kidney tissue from independent cohorts. ADAM12, TIM-3, IL-12p40, and CEACAM-5 were expressed in kidney tissue. A linear relationship was observed between the pathological grade of IgAVN and the expression levels of ADAM12 and CEACAM-5. Furthermore, while the prognosis of children with IgAVN may have a linear relationship with CEACAM-5, the results did not indicate a significant statistical difference, which may be related to the sample size. The expression of ADAM12 and CEACAM-5 were positively correlated with the pathological grade. More importantly, we found that CEACAM-5 may be related to the prognosis of IgAVN, which could serve as a significant biomarker for assessing disease severity and monitoring disease progression.

## Introduction

IgA vasculitis (IgAV) is the most common vasculitis in children and has been recognized for over 200 years. It is a systemic disease that affects multiple organs, typically involving the skin, gastrointestinal tract, joints, and kidneys [1, 2]. The annual incidence rate of IgAV ranges from 6.21 to 55.5 per 100,000 in different countries [3], with significant variation among different ethnic groups. Gardner-Medwin et al. [4] reported that in a large population-based survey, the incidence rate of IgAV among Asians, whites, and blacks was 20.4, 17.8, and 6.2 per 100,000 people per year, respectively.

The course of IgAV is mostly benign and self-limited, but renal damage caused by IgAV, known as IgAV with nephritis (IgAVN), can lead to chronic kidney disease and end-stage renal failure (ESRD) [5]. Renal involvement is the primary determinant of prognosis, as 20%–80% of children exhibit signs of nephritis, such as hematuria and/or proteinuria, within four to six weeks of the initial consultation. Furthermore, 1%–7% of children with IgAVN may progress to renal failure or ESRD [3, 6]. However, the pathological process and mechanisms leading from IgAV to IgAVN remain unclear. These mechanisms may involve immune abnormalities, genetic factors, abnormal coagulation mechanisms, complement factors, and neutrophil infiltration, accompanied by vascular inflammation [7, 8]. Recently, a multihit hypothesis has been proposed to explain the pathogenesis of IgAV and glomerulonephritis [8].

Renal biopsy is the gold standard for assessing renal injury in IgAVN, but it is invasive and cannot be used for dynamic monitoring. Moreover, several studies suggest that pathological results do not always correlate with final prognosis [9, 10]. Thus, the search for non-invasive biomarkers with high sensitivity and specificity is clinically important for early identification, timely intervention, dynamic monitoring, and evaluation of disease efficacy and prognosis [7]. Urinary biomarkers, such as urinary IgA levels [11], kidney injury molecule-1 (KIM-1), monocyte chemoattractant protein-1 (MCP-1), N-acetyl-β-glucosaminidase (NAG), and urinary angiotensinogen (UAGT) [12], appear to be related to the presence and/or severity of IgAVN. Another study indicated that the presence of IgA nephritis is associated with serum Gd-IgA1 and urinary IgA, IgG, IgM, IL-6, IL-8, IL-10, and IgA-IgG and IgA-sCD89 complexes [13], as well as neutrophil count, neutrophil/lymphocyte ratio [14], and serum angiotensinogen concentration [15]. However, reliable predictive biomarkers and core prognostic indicators are still lacking, necessitating further research.

Particularly, the serum contains various proteins released by diseased kidney tissue, highlighting the urgent need to study cytokines in IgAVN serum. The renal surveillance phase during childhood provides an ideal opportunity for early intervention. To date, there has been no research on serum-based high-throughput cytokines for IgAV and IgAVN.

Since the emergence of protein chip technology at the end of the last century, it has become a mature and stable technology in proteomics research, thanks to over 20 years of continuous development. A cytokine chip can detect the levels of various cytokines in serum or other body fluids with high sensitivity and specificity. Thus far, no studies have been conducted on IgAV and IgAVN using serum high-throughput cytokine analysis. This study aims to analyze IgAV and IgAVN based on serum cytokine levels, identify differentially expressed cytokines, enhance understanding of the IgAV to IgAVN transition, and provide insights into IgAVN pathogenesis. Additionally, it seeks to offer clues for future screening of molecular markers, monitor the occurrence, progression, and prognosis of IgAVN, and ultimately improve survival rates for patients with IgAVN.

## Materials and methods

### Patients, sample collection, and sample preparation

The validated EULAR/PRINTO/PRES criteria for diagnosing IgAV were used [16]. Renal pathological findings were classified according to the ISKDC classification. A follow-up period of more than six months was required for inclusion.

Serum samples from glucocorticoid-free cohorts of eight IgAV patients (G2) without severe other organ complications, eight IgAVN patients (G3), and eight normal patients without infections as the control group (G1) were used for the 440-plexed protein array screen. Serum samples were obtained from the Children’s Hospital of Zhejiang University School of Medicine. The agglutinated blood was centrifuged at 2000 rpm for 10 min. The upper serum was aliquoted at 500 µL per tube and frozen at −80 ^∘^C for later use.

The remaining paraffin samples from 18 IgAVN patients with renal puncture at different pathological levels were selected as the disease group (see Table 1 for details), and five normal renal specimens (hematuria with minor pathological changes) were selected as a control group. The pathological grade of IgAVN was based on the ISKDC standard (grades I–VI). Grade II was designated as the mild damage group (*n* ═ 6), and grades III–VI were designated as the moderate/severe damage group (*n* ═ 12). After standardized treatment (without cyclosporine A), children with normal physical signs, urine tests, and renal function were defined as the complete remission group (*n* ═ 8). However, the persistent abnormality group (*n* ═ 10) was characterized by hematuria (>5 red blood cells/high-power field or red blood cells casts or ≥2+ on dipstick) and/or proteinuria (>0.3 g/24 h or >30 mmol/mg of urine albumin:creatinine ratio) in a first-morning urine sample, persisting for >3 months.

**Table 1:** Clinical data of 18 IgAVN patients for validation of differential proteins

**Gender**	**Age**	**Urinary RBC (/HP)**	**24-h urinary protein (mg)**	**Serum creatinine (µmol/L)**	**ISKDC grade**	**Prognosis**
Male	12	131	169.00	54.00	IIa	A
Female	12	77	79.70	57.00	IIa	A
Male	10	170	1171.80	62.00	IIa	A
Female	10	>200	644.80	47.00	IIa	A
Female	14	7	1578.20	88.00	IIb	C
Male	13	12	1077.20	69.00	IIa	C
Female	14	24	593.30	74.00	IIIb	A
Male	15	>200	843.30	83.00	IIIb	A
Male	11	198	89.30	75.00	IIIa	B
Male	9	120	3396.80	94.00	IIIb	C
Male	12	9	6149.70	87.00	IIIb	C
Female	12	>200	1833.00	73.00	IIIb	A
Female	8	24	3685.30	69.00	VI	B
Female	16	>200	7474.60	82.00	IVb	B
Female	13	87	772.00	51.00	IVb	B
Male	10	>200	2557.40	87.00	IVb	A
Male	8	120	738.00	53.40	IIIb	C
Male	11	25	3080.60	69.00	IVb	C

IgAVN: IgA vasculitis with nephritis.

### Cytokine chip

A total of 440 cytokine expressions were measured using the GSH-CAA-440 kit. Operating procedures: Add 100 µL of sample diluent buffer to each well and incubate for 1 h on a shaker at room temperature (RT) to block the quantitative antibody chip. Remove the buffer, add 70 µL of sample to the wells, and incubate overnight at 4 ^∘^C. Slides were washed in two steps using the Thermo Scientific Well Wash Versa chip washer. Centrifuge the antibody mixture tubes, add 1.4 mL of sample diluent, mix evenly, and centrifuge again. Add 80 µL of detection antibody to each well and incubate for 2 h on a shaker at RT. Centrifuge the Cy3-streptavidin tube, add 1.4 mL of sample diluent, mix evenly, and centrifuge again. Add 80 µL of Cy3-streptavidin to each well and incubate for 1 h on an RT shaker in the dark (cover the slides with aluminum foil). Use a laser scanner (InnoScan 300) for fluorescence detection, taking the Cy3 or green channel (excitation frequency of 532 nm). Raybiotech software was used to subtract the chip background from the scanned raw data and normalize the data.

### Semiquantitative immunofluorescence analysis

First, paraffin sections of kidney tissue were dewaxed and rehydrated for antigen retrieval, then circled with a histochemical pen. Autofluorescence was quenched for 5 min and washed with running water for 10 min. The serum was then blocked for 30 min. Add the primary antibody, incubate at 4 ^∘^C overnight, then add the secondary antibody and incubate at RT for 50 min. Add the second primary antibody, incubate at 4 ^∘^C overnight in a wet box, and then add the second secondary antibody. Incubate for 50 min at RT in the dark. Finally, counterstain the nuclei with DAPI, incubate for 10 min at RT in the dark, cover the slides, and store them in a dark sectioning box at 4 ^∘^C.

Under ultraviolet light excitation, nuclei stained with DAPI appear blue, while fluorescein labeling appears red or green (considered positive). Image-Pro Plus 6.0 software (Media Cybernetics, Inc., Rockville, MD, USA) was used to select the same red or green colors, applying a unified positive standard to analyze the positive area, tissue area, and positive rate of each photo. Magnifications were 20.0X for CY3 and 20.0X-2 for FITC. The staining was classified into four grades: negative (−) for no positive staining; weak positive (+) for 0%–25% positive range; positive (++) for 26%–50% positive range; and strong positive (+++): positive range is greater than 50%.

### Principal component analysis (PCA)

PCA, a dimension reduction technique, converts multiple indicators into a few comprehensive indicators. We selected the first two principal components and plotted the distribution of samples on a two-dimensional plane based on their principal component scores. Outliers distant from most sample points were identified in the graph. PCA was performed using the prcomp function and visualized with the autoplot function in the ggfortify package from R/GitHub.

### Cluster heatmap

Hierarchical clustering and heatmaps were generated using the heatmap.2 function from the gplots package in R. Euclidean distance was used to calculate the distance between samples, and the furthest neighbor method (complete linkage clustering) was employed to calculate the distance between clusters.

### Gene Ontology (GO)

GO is an international standard classification system for gene function, comprising three parts: molecular functions (MFs), biological processes (BPs), and cellular components. GO analysis was performed using the Fisher exact test with the clusterProfiler package from R/Bioconductor. Selection criteria were: ① at least two differential proteins falling under one GO term and ② sorting by count in descending order and selecting the top ten outcomes.

### Kyoto Encyclopedia of Genes and Genomes (KEGG) pathway enrichment

KEGG is a database for systematically analyzing gene function and genomic information, studying gene and expression data within network contexts. It provides pathways for carbohydrates, amino acids, and nucleosides, while comprehensively annotating enzymes catalyzing each reaction step. KEGG pathway enrichment analysis helps identify biological regulatory pathways enriched by differentially expressed genes.

### Ethical statement

The study was approved by the ethics committee of the Children’s Hospital of Zhejiang University School of Medicine (2021-IRB-118).

### Statistical analysis

Data were plotted and analyzed using GraphPad Prism V.7 (GraphPad, San Diego, CA, USA), Microsoft Excel, or R. The Mantel–Haenszel chi-square test was used for semiquantitative analysis of immunofluorescence for selected differential cytokines. Pearson’s correlation coefficient was used to assess correlations between variables. Differences were considered statistically significant at *P* < 0.05.

Processed cytokine chip data were analyzed using moderated *t*-statistics with the R/Bioconductor limma package. Differential proteins were selected by adjusting the *P* value (BH-adjusted *P* value, or *q* value) and the log FC (log fold change with base 2), and the selection conditions were as follows: (1) log FC > 1.2 or <0.83; (2) Adjust the *P* value (or the *q* value) < 0.05; and (3) It is recommended to select the average (fluorescence) signal value of each group *P* > 150.

**Table 2:** 81 differential proteins in the control group (G1) and IgAV group (G2)

**ProteinID**	**AveExp.G2**	**AveExp.G1**	***P* val**	**Foldchange**	**Regulation**
IL-5 Ra	8.529398836	7.779048688	1.84E-09	1.682201058	up
IL-1 F10	8.654393215	7.933092859	2.57E-08	1.648667371	up
Dkk-4	8.50631367	7.75655004	2.32E-07	1.68151731	up
PSMA	7.536844644	6.833265305	3.76E-07	1.628540207	up
CTLA4	10.10294847	9.184315353	6.44E-07	1.890323451	up
FGF-9	8.226036603	7.312374756	7.05E-07	1.883820947	up
IL-10 Ra	7.876063727	7.008600192	8.21E-07	1.82445243	up
CA9	8.551081888	7.466363683	1.01E-06	2.120961164	up
IL-1 F7	7.893183998	7.045198708	1.13E-06	1.799985506	up
IL-1 R5	8.324550891	7.599483893	2.78E-06	1.652977398	up
IL-1 F6	7.870417921	7.121807239	3.79E-06	1.680174041	up
Mer	8.345026492	7.665905849	8.60E-06	1.60116351	up
Prostasin	8.627650628	8.077029191	1.83E-05	1.464716482	up
IGFBP-5	7.988724219	7.266678841	4.31E-05	1.64951898	up
CD14	16.97372479	16.53037533	0.000383856	1.359757566	up
FOLR1	8.495195303	9.248988579	0.000535636	0.593042221	down
Epo R	7.421433748	6.397421702	0.00053777	2.033566326	up
Gas 1	7.732922262	7.197492999	0.00055622	1.44937334	up
SIGIRR	8.518055554	8.067164119	0.000623094	1.366884589	up
CD6	10.82325501	11.47029304	0.001052404	0.638590046	down
Cadherin-13	13.0610857	14.53960655	0.001205881	0.358856549	down
EDA-A2	7.459502711	6.508260482	0.002016796	1.933536809	up
IL-1 F9	7.443447297	6.437414335	0.002085197	2.008380972	up
IL-17C	7.201885813	5.512816057	0.002200848	3.22448723	up
FGF-6	7.859994974	7.234509717	0.002384407	1.542729642	up
MMP-3	13.29195803	11.88050492	0.003068949	2.660049537	up
sFRP-3	12.51070164	12.87476102	0.003462739	0.77697529	down
Thrombospondin-5	16.71859078	17.47711604	0.003271833	0.59110025	down
IL-18 BPa	11.35550633	10.99622958	0.003473132	1.282782651	up
Contactin-2	10.85373962	11.44302996	0.004091963	0.664669778	down
IL-1 F8	8.706353522	8.190450581	0.004729668	1.429888786	up
IL-1 F5	8.464808353	7.757934707	0.005506973	1.632263129	up
Thrombomodulin	18.43627358	18.71452753	0.005794607	0.824588388	down
ANG-4	7.994817845	8.543772546	0.006289324	0.683515187	down
Follistatin-like 1	12.52279263	11.89615242	0.007202316	1.543965174	up
IL-1a	9.275109099	9.804984507	0.007870924	0.692614546	down
IL-5	9.214937166	9.503289838	0.007980646	0.818836505	down
Ferritin	16.41287519	16.86049236	0.008572211	0.733252928	down
G-CSF R	9.58176046	10.53390573	0.008704336	0.51686332	down
SDF-1a	10.16894845	9.774210167	0.008690632	1.314704244	up
VEGF R2	14.00558547	14.83284388	0.009257633	0.563599248	down
TIM-3	12.15264921	12.92353405	0.010991748	0.58605792	down
ULBP-1	8.955937864	8.385779706	0.010845527	1.484686323	up
Leptin R	7.475971541	6.66756798	0.012915235	1.751272469	up
IL-1 RII	12.46330657	13.42036189	0.01346337	0.515107226	down
NOV	16.84288996	17.35733522	0.013434005	0.700062064	down
LIGHT	10.85605337	10.52533702	0.014275118	1.257637687	up
EG-VEGF	11.19444261	11.89827575	0.014122662	0.613938848	down
Layilin	8.219311419	7.628073574	0.015297551	1.506538817	up
Clusterin	8.787114732	9.374755635	0.016936871	0.66543013	down
TWEAK	7.8781836	7.456879235	0.019038317	1.339137745	up
GITR	12.26247888	11.84935479	0.019594571	1.33156614	up
I-309	8.24105491	8.709020702	0.020220397	0.72298329	down
CD97	12.56719894	13.01031656	0.021107689	0.735543404	down
HAI-2	9.115695743	9.800123274	0.020847738	0.622252691	down
MIP-1d	16.52831974	17.09600142	0.022595896	0.674700118	down
RANK	9.163350759	9.880664369	0.025030234	0.608228949	down
Angiotensinogen	13.19961602	11.46258796	0.026555558	3.333477669	up
CD58	11.06890865	9.908931329	0.027139596	2.234539147	up
MIP-3a	8.773137524	8.471090312	0.026948485	1.232892671	up
TACI	10.13116866	10.86420325	0.028168278	0.601637088	down
CF XIV	11.58614057	12.43379176	0.028287896	0.555688703	down
Fractalkine	11.01014618	11.52462173	0.028999981	0.700047372	down
CXCL14	14.98953843	14.71800592	0.032004773	1.20708938	up
TIMP-1	15.20477588	15.51449558	0.033654234	0.806798493	down
Albumin	16.80125753	17.36846128	0.034261527	0.67492367	down
TF	7.434139948	6.453278497	0.035603039	1.973643545	up
Aggrecan	11.70402271	12.09771274	0.035035894	0.761180215	down
Tie-1	10.89391634	11.49239428	0.035194351	0.660450369	down
Prolactin	13.96213724	13.09516958	0.036637991	1.823825453	up
XEDAR	11.50118611	11.94212303	0.038011973	0.736656049	down
CD40	8.236020572	7.852693904	0.042229617	1.304346044	up
IL-20	6.570112245	5.064887048	0.041642072	2.838689783	up
Cystatin A	11.74190867	11.13137993	0.04139414	1.526818672	up
MIG	7.711830903	8.274656525	0.042646043	0.67697496	down
IL-2 Rg	9.262331099	9.813714992	0.041277217	0.682365261	down
CA19-9	11.0684249	11.40710723	0.043259477	0.790763218	down
Adiponectin	18.48046969	18.18175367	0.046071249	1.2300492	up
AFP	13.24487909	13.69842743	0.046138475	0.730244586	down
VEGF-C	8.499474129	8.139240646	0.048135088	1.283633622	up
Furin	13.53645704	14.20489169	0.049685185	0.629189002	down

IgAV: IgA vasculitis.

**Figure 1.**
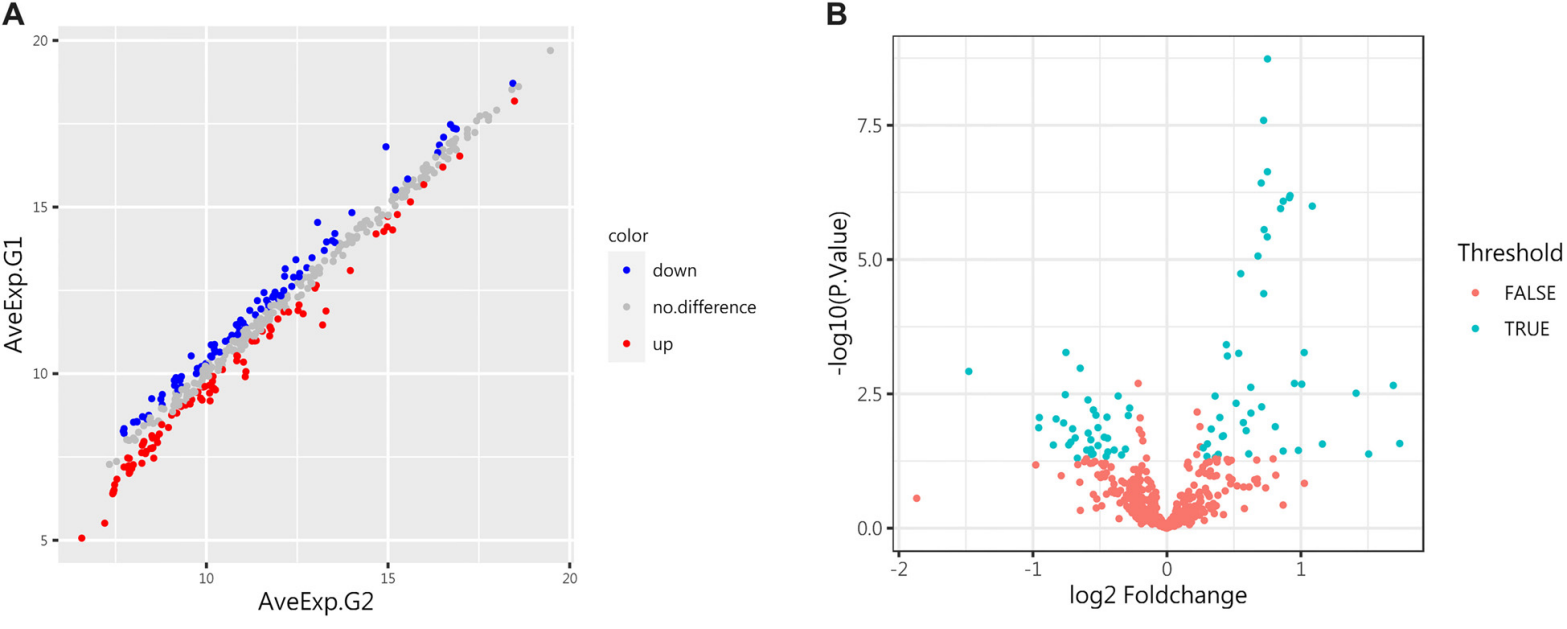
**Differentially expressed proteins (DEPs) in the control group (G1) and the IgAV group (G2).** (A) The scatter plot displays the expression of all proteins in both groups. Red indicates upregulation, blue indicates downregulation, and gray indicates no significant difference. (B) The volcano plot highlights proteins with significant differences between the control and IgAV groups, showing 36 downregulated and 45 upregulated proteins (*P* < 0.05). IgAV: IgA vasculitis.

**Figure 2.**
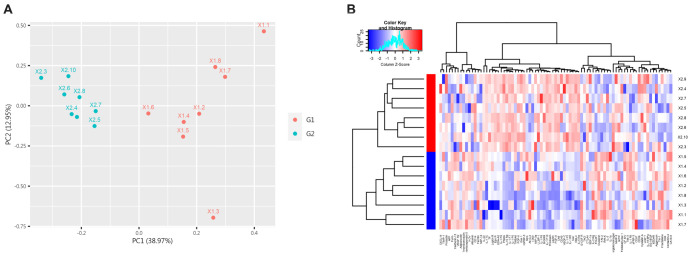
**PCA and clustering heatmap of all DEPs in the control group and the IgAV group.** (A) The first two principal components are plotted to illustrate the differences between the two groups. A smaller distance between points indicates greater similarity, while a larger distance indicates greater dissimilarity. (B) Euclidean distance and complete linkage clustering were used to analyze the dissimilarities between the two groups. Red represents G2, and blue represents G1. DEP: Differentially expressed proteins; IgAV: IgA vasculitis; PCA: Principal component analysis.

## Results

### Protein array screen between IgAV group and normal control group

#### Differential protein screening between IgAV group and normal control group

According to the screening criteria, a total of 81 proteins showed significant differences between the IgAV group (G2) and the control group (G1) (adjust *P* value<0.05, log FC > 1.2 or<0.83), as shown in Table 2 of the attachment. Differential proteins are represented by scatter plots and volcano plots, as illustrated in Figure 1. Compared to the normal control group, the expression of 45 proteins increased in the IgAV group, such as CA9, Epo R, IL-1 F9, IL-17C, MMP-3, Angiotensinogen, CD58, IL-20, etc. Meanwhile, the expression of 36 proteins was downregulated, including Cadherin-13, G-CSF R, VEGF R2, TIM-3, IL-1 RII, and CF XIV.

**Figure 3.**
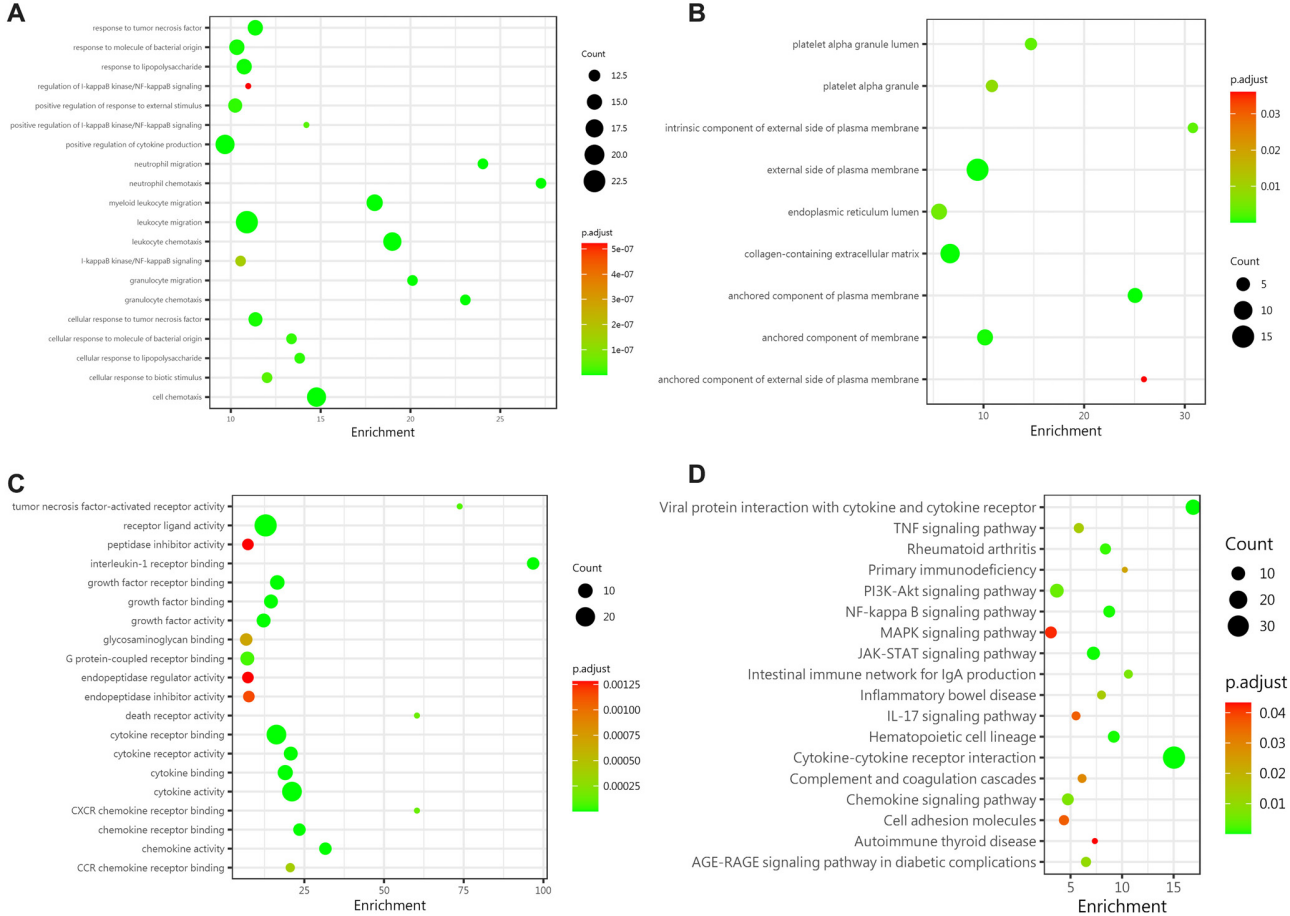
**Biological analysis of differential proteins between the IgAV group and the normal control group.** (A) BPs showed that the differences between the two groups mainly involved leukocyte chemotaxis, leukocyte migration, positive regulation of cytokine expression, and responses to biological stimulation, bacterial molecules, and tumor necrosis factors; (B) In terms of CC, the differential proteins were primarily located outside the plasma membrane and within the ECM; (C) Differences in MF mainly focused on carboxylic acid binding, organic acid binding, transmembrane receptor protein tyrosine kinase activity, hyaluronic acid binding, and amide binding; (D) KEGG pathway enrichment analysis revealed that the most significantly altered pathways between the two groups were cytokine–cytokine receptor interaction, viral protein interaction with cytokine and cytokine receptor, JAK-STAT, hematopoietic cell lineage, NF-kappa B, and PI3K-Akt signaling. IgAV: IgA vasculitis; BPs: Biological processes; CC: Cellular composition; ECM: Extracellular matrix.

PCA and cluster heatmap analysis were performed on all proteins between the two groups. PCA of the 81 differential proteins was conducted using the prcomp function in R software. The first and second principal components (PC1 and PC2) were selected and plotted to show the differences. Figure 2A shows that these two principal components can effectively distinguish the IgAV group from the normal control group. The differences between the two groups were further analyzed using Euclidean distance and complete clustering, as shown in Figure 2B.

#### Biological pathway analysis of differential proteins between IgAV group and normal control group

To further explore the pathogenesis of IgAV and understand the biological significance of differentially expressed proteins, we performed GO enrichment analysis and KEGG enrichment analysis. A total of 411 GO terms were enriched. The results of the differential protein biological analysis were as follows: (1) In terms of BPs, differences were mainly concentrated in leukocyte chemotaxis, leukocyte migration, positive regulation of cytokine expression, and responses to biological stimuli, bacteria-derived molecules, and tumor necrosis factor (Figure 3A); (2) In terms of cellular composition (CC), differences were primarily located outside the plasma membrane and extracellular matrix (ECM) (Figure 3B); and (3) For MF, differences were focused on carboxylic acid binding, organic acid binding, transmembrane receptor protein tyrosine kinase activity, hyaluronic acid binding, and amide binding (Figure 3C). KEGG pathway enrichment analysis revealed 18 significantly different signaling pathways, including cytokine–cytokine receptor interaction, viral protein interaction with cytokines, JAK-STAT, hematopoietic cell lineage, NF-kappa B, and PI3K-Akt (Figure 3D).

### Protein array screen between IgAVN group and normal control group

#### Differential protein screening between IgAVN group and normal control group

**Figure 4.**
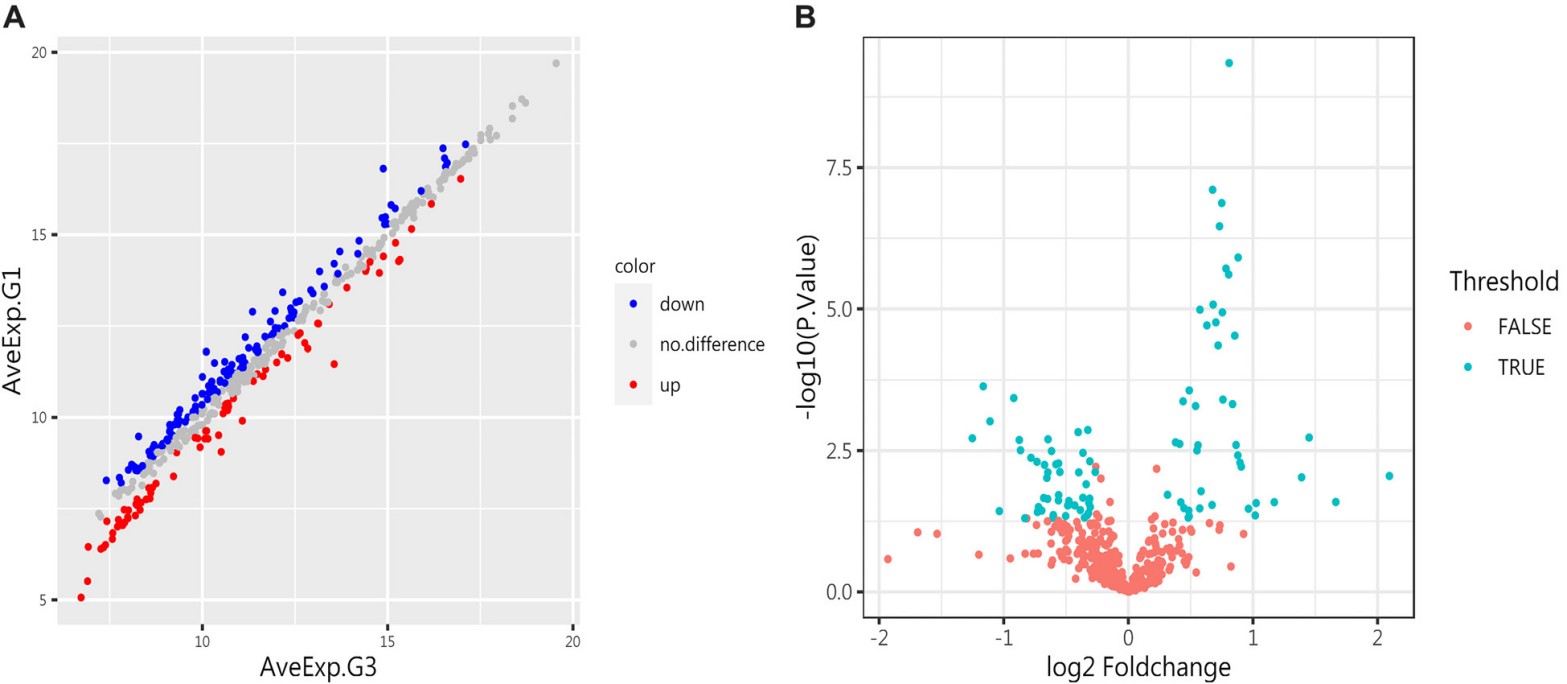
**Differentially expressed proteins (DEPs) in the control group and the IgAVN group (G3).** (A) The scatter plot illustrates protein expression in both groups. Red indicates upregulation, blue indicates downregulation, and gray indicates no significant difference. (B) The volcano plot shows the proteins with significant differences between the two groups. A total of 44 proteins were upregulated, and 48 proteins were downregulated (*P* < 0.05). IgAVN: IgA vasculitis with nephritis.

To investigate the pathogenesis of IgAVN, we compared the IgAVN group (G3) with the normal control group (G1). A total of 92 proteins were significantly different (adjust *P* value<0.05, logFC > 1.2 or<0.83), as shown in Table 3 of the attachment. Compared to the control group, 44 proteins were upregulated and five were downregulated in the IgAVN group. See Figure 4 for details.

**Figure 5.**
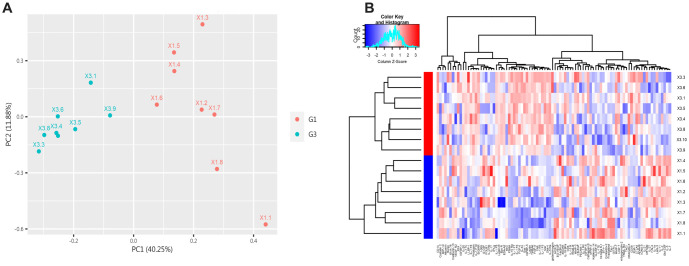
**PCA and clustering heatmap of all DEPs in the control group and the IgAVN group.** (A) The first two principal components are plotted to illustrate the differences between the two groups. A smaller distance between points indicates greater similarity, while a larger distance indicates greater dissimilarity. (B) Clustering heatmap of the differential proteins, where red represents G3, and blue represents G1. IgAVN: IgA vasculitis with nephritis; PCA: Principal component analysis.

PCA and cluster heatmap analyses were conducted to examine the differential proteins between the two groups. Figure 5A shows that these two principal components can effectively distinguish the IgAVN group from the normal control group. The differences between the two groups were analyzed using Euclidean distance and complete clustering, as shown in Figure 5B.

**Table 3:** 92 differential proteins in the control group (G1) and IgAVN group (G3)

**ProteinID**	**AveExp.G3**	**AveExp.G1**	***P* val**	**Foldchange**	**Regulation**
IL-5 Ra	8.587772936	7.779048688	4.48E-10	1.751661791	up
IL-1 F10	8.609208256	7.933092859	7.81E-08	1.597831633	up
PSMA	7.582704035	6.833265305	1.34E-07	1.681138669	up
Dkk-4	8.48800808	7.75655004	3.46E-07	1.660316219	up
FGF-9	8.193540641	7.312374756	1.23E-06	1.841863165	up
IL-1 F6	7.905375065	7.121807239	1.93E-06	1.721382642	up
IL-1 F7	7.850183279	7.045198708	2.46E-06	1.747127107	up
Mer	8.34617964	7.665905849	8.40E-06	1.602443834	up
Prostasin	8.651627667	8.077029191	1.03E-05	1.48926292	up
CTLA4	9.939025096	9.184315353	1.15E-05	1.687292091	up
IL-10 Ra	7.71074225	7.008600192	1.73E-05	1.626918586	up
IL-1 R5	8.229726797	7.599483893	1.96E-05	1.547825576	up
CA9	8.320246892	7.466363683	2.98E-05	1.807359137	up
IGFBP-5	7.987101262	7.266678841	4.43E-05	1.6476644	up
Tie-1	10.32799165	11.49239428	0.000230573	0.446148956	down
SIGIRR	8.55588922	8.067164119	0.000272174	1.403204326	up
Fractalkine	10.60491526	11.52462173	0.00037156	0.528616562	down
FGF-6	7.993483024	7.234509717	0.000394889	1.692285881	up
CD14	16.96942856	16.53037533	0.000424392	1.355714342	up
ULBP-1	9.221997293	8.385779706	0.000475381	1.78536319	up
Gas 1	7.737160113	7.197492999	0.000514026	1.453637068	up
IL-17E	10.00249298	11.11257013	0.000958802	0.463269254	down
IL-11	8.906543704	9.231780792	0.001364559	0.798167208	down
sFRP-3	12.47190032	12.87476102	0.001488822	0.756357026	down
IL-1 RII	12.16826708	13.42036189	0.001912104	0.419838155	down
Albumin	16.49322831	17.36846128	0.002050573	0.545165826	down
CEACAM-5	10.50843932	9.057754762	0.001856233	2.733377187	up
Contactin-2	10.79863947	11.44302996	0.001991088	0.639763023	down
IL-18 BPa	11.37520466	10.99622958	0.002255265	1.300417686	up
Epo R	7.261786324	6.397421702	0.002514344	1.820537706	up
IL-1 F8	8.749119551	8.190450581	0.002536912	1.472909684	up
MMP-7	10.70798865	10.29600402	0.002406281	1.330514866	up
TWEAK	8.007753991	7.456879235	0.003138789	1.464973692	up
I-309	8.092436862	8.709020702	0.003219927	0.652213478	down
MIG	7.409234685	8.274656525	0.003119782	0.548885891	down
IL-17	8.59730656	8.961491896	0.003463738	0.776907457	down
EDA-A2	7.386954528	6.508260482	0.003832658	1.838710114	up
PECAM-1	11.83721828	12.61864811	0.004231785	0.581789907	down
Thrombospondin-2	10.25255934	10.98562933	0.004986915	0.601622324	down
IL-5	9.194176184	9.503289838	0.004879392	0.807137486	down
IL-1 F9	7.333781039	6.437414335	0.005177028	1.861372378	up
IL-1a	9.244825304	9.804984507	0.005324926	0.678227316	down
IL-27	9.138067051	9.719172604	0.00552399	0.668451339	down
GITR L	10.58623288	11.25884766	0.005654972	0.627368597	down
Leptin R	7.573717903	6.66756798	0.006073925	1.874037631	up
FOLR1	8.699472056	9.248988579	0.007544918	0.683249061	down
TRAIL R2	10.00398908	10.65004062	0.007676725	0.639026851	down
IL-15 R	10.81217824	11.20985229	0.007673561	0.75908111	down
IFNg	8.673464253	8.938925862	0.007566621	0.831932504	down
Angiotensinogen	13.55751716	11.46258796	0.00888036	4.272051982	up
IL-17C	6.902969408	5.512816057	0.009379759	2.621065399	up
Ck beta 8-1	9.993514904	10.64724391	0.009601783	0.635635229	down
IL-7	9.183026615	9.521595477	0.012452408	0.790825413	down
Layilin	8.211364501	7.628073574	0.016554195	1.498263037	up
Kallikrein 14	12.62158163	13.18109769	0.019317065	0.678529732	down
LIGHT	10.83978935	10.52533702	0.019106457	1.243539501	up
Cystatin C	16.60542714	16.97007649	0.021556872	0.776657619	down
HAI-2	9.120941175	9.800123274	0.021737422	0.62451923	down
Nectin-4	11.50891384	11.81930345	0.02233675	0.806423949	down
EG-VEGF	11.25041533	11.89827575	0.022589804	0.638226131	down
IL-20	6.729615236	5.064887048	0.025628185	3.170539143	up
IL-34	7.892387994	7.471750554	0.025902222	1.338518835	up
B7-H1	10.31323476	10.79116941	0.024533866	0.718004781	down
CD58	11.07997582	9.908931329	0.025850333	2.251746615	up
MIP-1d	16.53505545	17.09600142	0.024072766	0.67785755	down
Renin	15.29608406	14.27082654	0.02676301	2.035322657	up
GM-CSF	9.059223199	9.375952064	0.026687904	0.802888267	down
XEDAR	11.47234545	11.94212303	0.027996513	0.722075909	down
IL-21	9.804620521	10.23497443	0.029427465	0.742079722	down
CRTAM	9.816235905	10.30271854	0.029493356	0.713763171	down
HB-EGF	12.30326112	11.63144347	0.028949643	1.593078822	up
IGFBP-1	15.09325038	15.81471844	0.031466367	0.606479985	down
IL-2	8.923021594	9.228681745	0.031507236	0.809071915	down
Cathepsin B	13.13425723	12.56136272	0.033279257	1.487504993	up
ADAM8	9.875674705	9.428992359	0.032965506	1.36290249	up
MMP-3	12.84435731	11.88050492	0.033725763	1.950511352	up
TACI	10.16887391	10.86420325	0.036399263	0.617568323	down
CD6	11.0846961	11.47029304	0.035674297	0.76546221	down
Pref-1	10.11561185	9.631406438	0.036496169	1.398815237	up
MCP-2	11.15782815	12.19281341	0.037136237	0.488020868	down
G-CSF R	9.804730885	10.53390573	0.038485927	0.603248845	down
RGM-B	10.88610964	11.20584376	0.040271689	0.801217524	down
IL-2 Rb	8.760493039	9.090557145	0.044000896	0.795501135	down
ENA-78	15.33246802	14.31412419	0.044311142	2.025592295	up
PF4	15.64460986	15.15703306	0.045770464	1.402087901	up
IGFBP-6	12.38328545	12.98508871	0.046103762	0.658929825	down
G-CSF	8.560145089	9.063239316	0.045422856	0.705591836	down
ICAM-3	9.323764197	9.925927402	0.043653379	0.658765449	down
LAG-3	9.051986195	9.403962513	0.048500065	0.783510048	down
IL-1 F5	8.238800105	7.757934707	0.048553249	1.395580552	up
Cadherin-13	13.70926922	14.53960655	0.049669321	0.562397729	down
VEGF R2	14.22793621	14.83284388	0.049186759	0.65751346	down

IgAVN: IgA vasculitis with nephritis.

#### Biological pathway analysis of differential proteins between IgAVN group and normal control group

**Figure 6.**
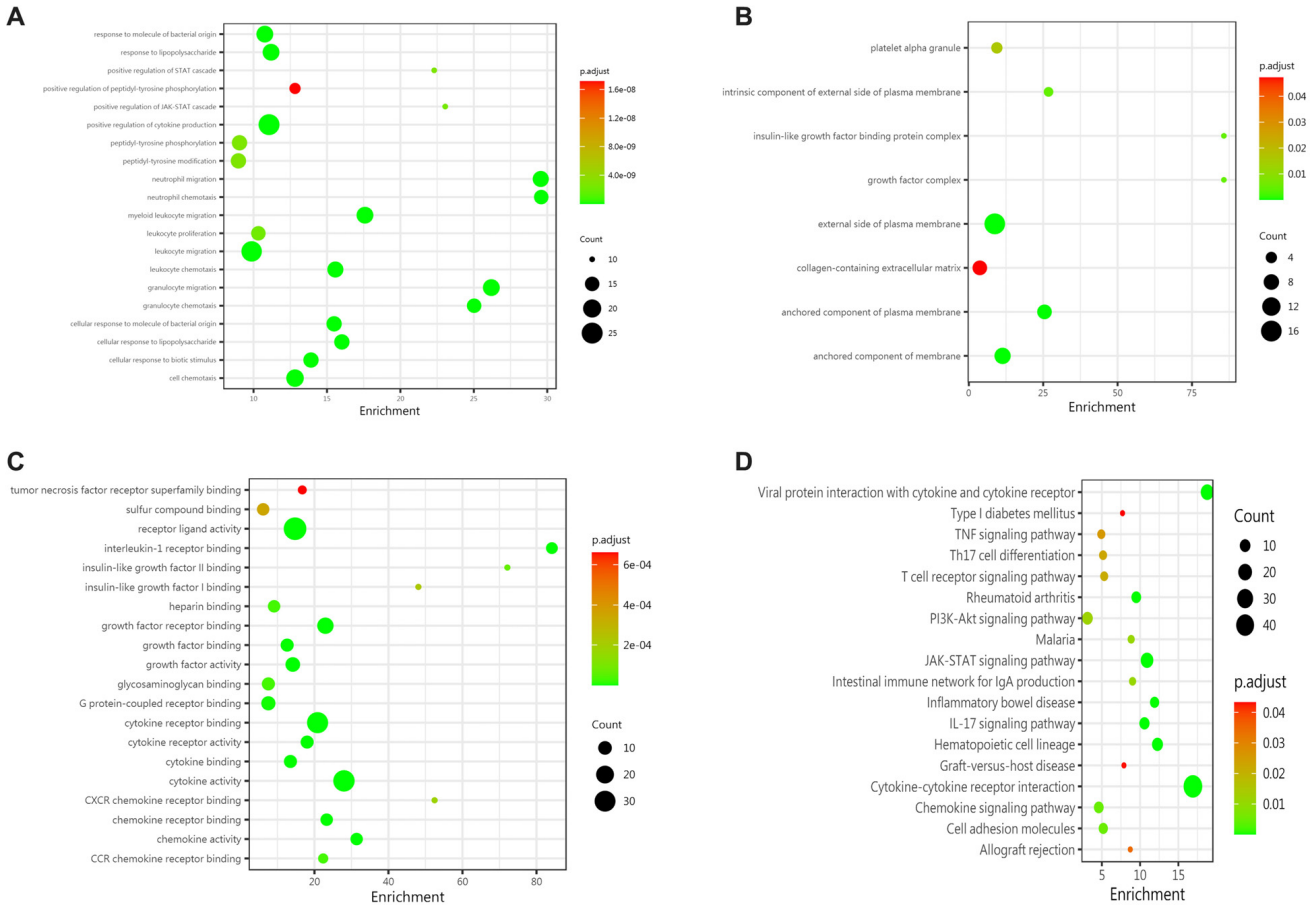
**Biological analysis of differential proteins between the IgAVN group and the normal control group.** (A) BPs showed that the differences between the two groups primarily involved leukocyte migration, chemotaxis, positive regulation of cytokine expression, response to biological stimulation, bacterial molecules, tumor necrosis factor, and tyrosine phosphorylation and modification; (B) In terms of CC, the differential proteins were mainly located on the outer side of the plasma membrane, cell membrane, and ECM; (C) Differences in MFs between the two groups were mainly related to cytokine activity, receptor–ligand activity, cytokine receptor binding, growth factor receptor binding, and interleukin-1 receptor binding; (D) The KEGG pathway enrichment analysis revealed that the most significantly altered signaling pathways between the two groups included cytokine–cytokine receptor interaction, viral protein interaction with cytokine and cytokine receptor, JAK-STAT, hematopoietic cell lineage, IL-17, rheumatoid arthritis, and PI3K-Akt pathways. IgAVN: IgA vasculitis with nephritis; BPs: Biological processes; CC: Cellular composition; MFs: Molecular functions; ECM: Extracellular matrix.

To better understand the biological significance of differentially expressed proteins, we performed GO and KEGG pathway analyses on the 44 differential proteins. Significant enrichment (adjusted *P* value < 0.05, FDR<0.05) resulted in 479 GO terms (Figure 6A–6C) and 18 pathways (Figure 6D). The biological analysis of proteins revealed the following: (1) BPs showed differences mainly in leukocyte migration, chemotaxis, positive regulation of cytokine expression, and responses to biological stimuli, bacteria-derived molecules, tumor necrosis factor, tyrosine phosphorylation, and modification (Figure 6A); (2) CC showed differences primarily located on the outer side of the plasma membrane, cell membrane, and ECM (Figure 6B); and (3) MF differences were concentrated in cytokine activity, receptor-ligand activity, cytokine receptor binding, growth factor receptor binding, and interleukin-1 receptor binding (Figure 6C). KEGG enrichment analysis showed significant enrichment in pathways such as cytokine–cytokine receptor interaction, viral protein interaction with cytokines, JAK-STAT, hematopoietic cell lineage, IL-17, rheumatoid arthritis, and PI3K-Akt (Figure 6D).

### Protein array screen of proteins between IgAVN group and IgAV group

#### Differential protein screening between IgAVN group and IgAV group

**Figure 7.**
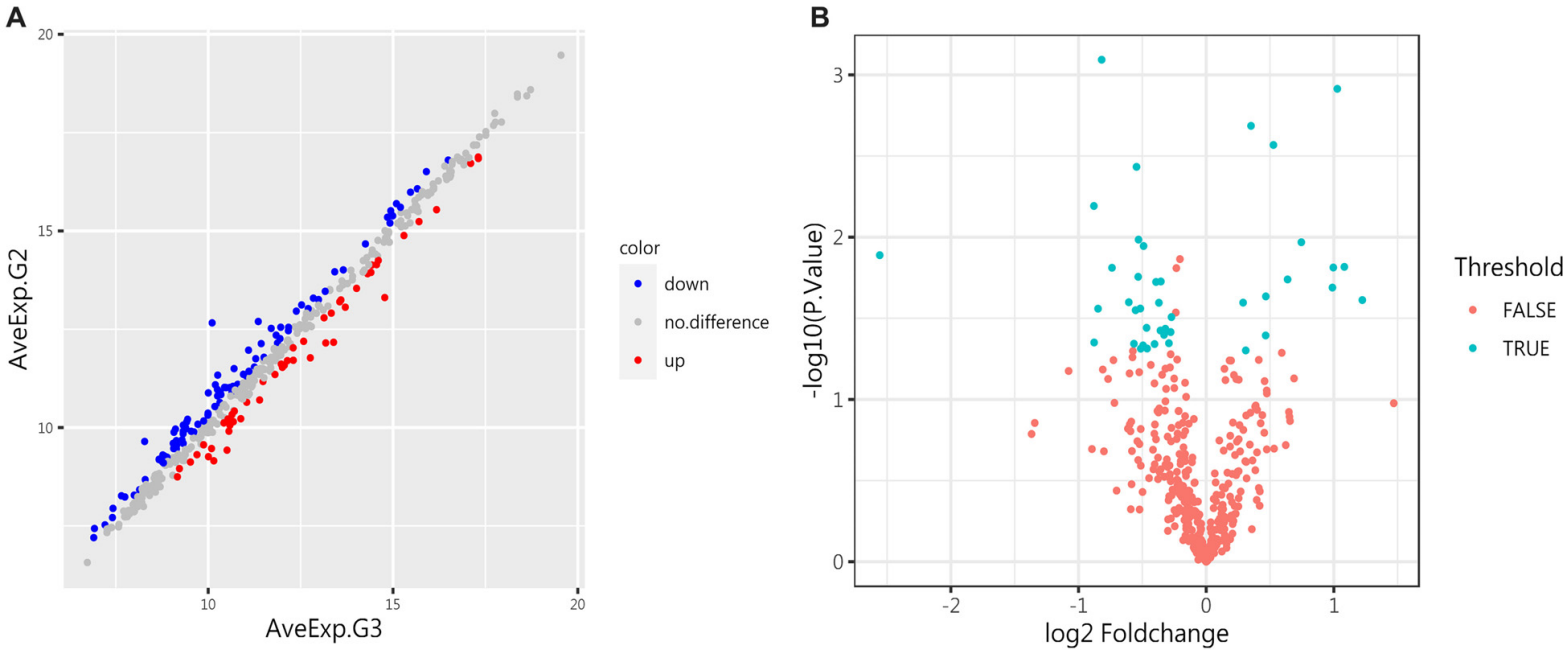
**Differentially expressed proteins (DEPs) between G2 and G3.** (A) The scatter plot shows protein expression in both groups. Red indicates upregulation, blue indicates downregulation, and gray indicates no significant difference. (B) The volcano plot highlights the proteins with significant differences between the two groups. Thirteen proteins were significantly upregulated, and 28 proteins were significantly downregulated (*P* < 0.05).

To further investigate the pathogenesis, we compared the IgAVN group (G3) with the IgAV group (G2). A total of 41 proteins were significantly different (adjusted *P* value<0.05, logFC > 1.2 or<0.83), as shown in Table 4 of the attachment. Compared to the IgAV group, 13 proteins were upregulated in the IgAVN group, including VEGF R3, ADAM12, TIM-3, CEACAM-5, and IL-12p40. Conversely, 28 proteins were downregulated, including Resistin, IL-17E, LRP-6, MCP-3, Follistatin-like 1, ICAM-3, TRAIL R3, Tie-1, and Thrombospondin-2. See Figure 7 for details.

**Table 4:** 41 differential proteins in IgAV group (G2) and IgAVN group (G3)

**ProteinID**	**AveExp.G3**	**AveExp.G2**	***P* val**	**Foldchange**	**Regulation**
Follistatin-like 1	11.70399889	12.52279263	0.000806488	0.56691575	down
TIM-3	13.1798669	12.15264921	0.001218737	2.038089896	up
DLL1	10.58660205	10.05980345	0.002703331	1.440728608	up
IGFBP-3	14.60217531	14.2509389	0.002061122	1.275653408	up
LAG-3	9.051986195	9.59768735	0.003687669	0.685058381	down
CD40	7.745569547	8.236020572	0.011338853	0.711802534	down
Resistin	10.10620877	12.66332365	0.012912277	0.169915001	down
VEGF R1	8.770583462	9.303286153	0.017546017	0.691258543	down
MMP-9	14.9918034	15.3847802	0.018876612	0.761556614	down
IL-27	9.138067051	9.668027153	0.010356552	0.692573887	down
IL-17E	10.00249298	10.88207685	0.006423093	0.54352418	down
Periostin	13.65542427	14.01044472	0.018757453	0.781858559	down
ADAM12	10.15176935	9.155765497	0.015381219	1.994467825	up
CEACAM-5	10.50843932	9.426099042	0.015240175	2.117468158	up
JAM-B	10.00587938	9.260418835	0.01074307	1.676509367	up
ICAM-3	9.323764197	10.0615773	0.015439856	0.599647634	down
RAGE	16.17815141	15.54033838	0.01821324	1.555968688	up
IL-2 Rb	8.760493039	9.130840497	0.025360324	0.773596162	down
Tie-2	8.671145363	9.186889773	0.027534264	0.699431942	down
NOV	17.3102671	16.84288996	0.023175053	1.382593593	up
MMP-7	10.70798865	10.41878264	0.025320236	1.221967575	up
Thrombospondin-2	10.25255934	10.80568075	0.028193181	0.681543942	down
MCP-3	9.114795591	9.961940756	0.02758364	0.555883642	down
VEGF R3	12.76272365	11.77377462	0.02044866	1.98473863	up
IL-12p40	13.39255651	12.16938504	0.024410073	2.334593669	up
TRAIL R3	15.90297251	16.50909408	0.02517885	0.656960454	down
LRIG3	10.63976066	10.91173525	0.031080073	0.82818525	down
AgRP	9.139694504	9.459679108	0.036670255	0.801078427	down
SDF-1b	8.814408394	9.277200479	0.048529934	0.725580664	down
ANGPTL3	11.48238118	11.17249103	0.049864119	1.239613318	up
LRP-6	11.09119302	11.96899667	0.0445571	0.544195278	down
Nectin-4	11.50891384	11.78719482	0.038442698	0.824572938	down
Tie-1	10.32799165	10.89391634	0.045361891	0.675522305	down
B7-H3	15.70396957	15.23803264	0.040331398	1.381214063	up
SDF-1a	9.877110028	10.16894845	0.045016392	0.81686047	down
IL-10	8.887569733	9.245942284	0.037594494	0.780044021	down
TNFb	9.063615118	9.467967611	0.045544285	0.755575332	down
IL-17R	11.28555988	11.75247294	0.036186111	0.723511041	down
MICA	10.34401746	10.83854077	0.046454787	0.709796168	down
PDGF Rb	11.10186542	11.43202518	0.039989732	0.795448391	down
PECAM-1	11.83721828	12.35001973	0.04864175	0.700860174	down

IgAV: IgA vasculitis; IgAVN: IgA vasculitis with nephritis.

**Figure 8.**
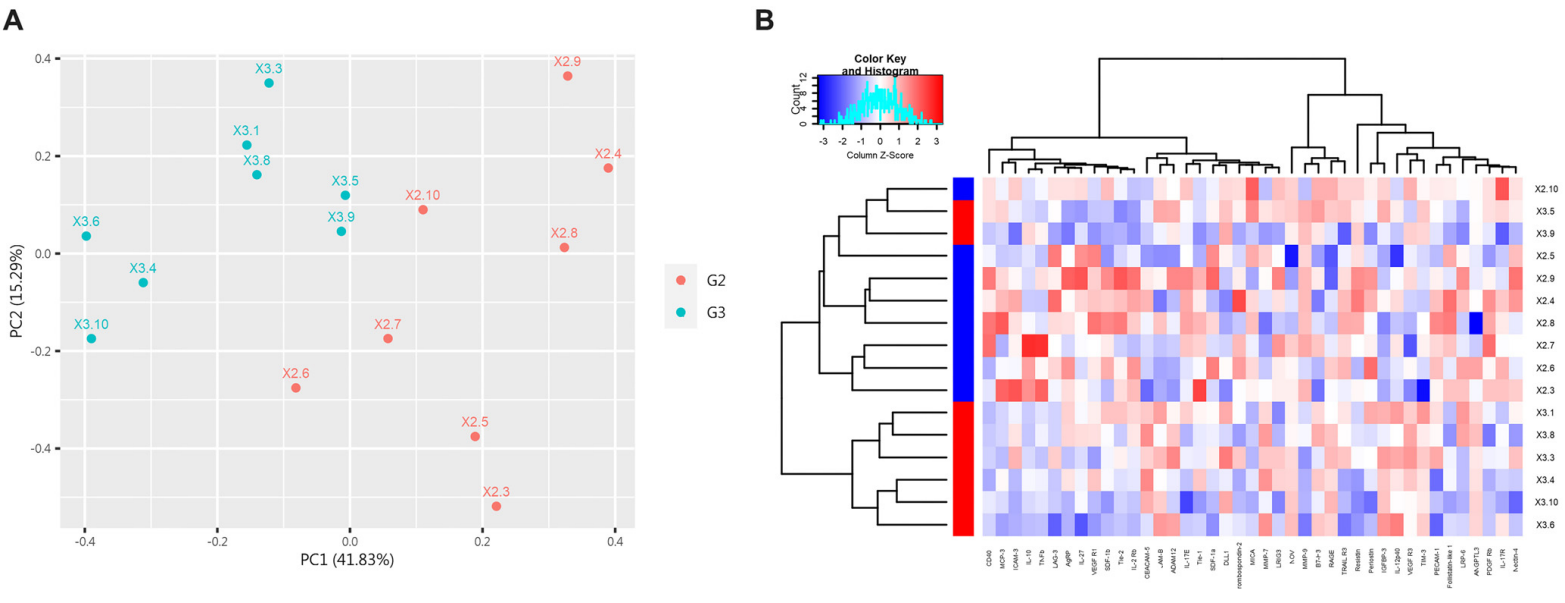
**PCA and clustering heatmap of all DEPs in G2 and G3.** (A) The first two principal components are plotted to illustrate the differences between the two groups. A smaller distance between points indicates greater similarity, while a larger distance indicates greater dissimilarity. (B) Cytokine clustering heatmap, with red representing G3 and blue representing G2. PCA: Principal component analysis.

PCA and cluster heatmap analyses were performed on all proteins between the two groups. The results, demonstrating distinguishable patterns, are shown in Figure 8A and 8B.

#### Biological pathway analysis of differential proteins between IgAVN group and IgAV group

**Figure 9.**
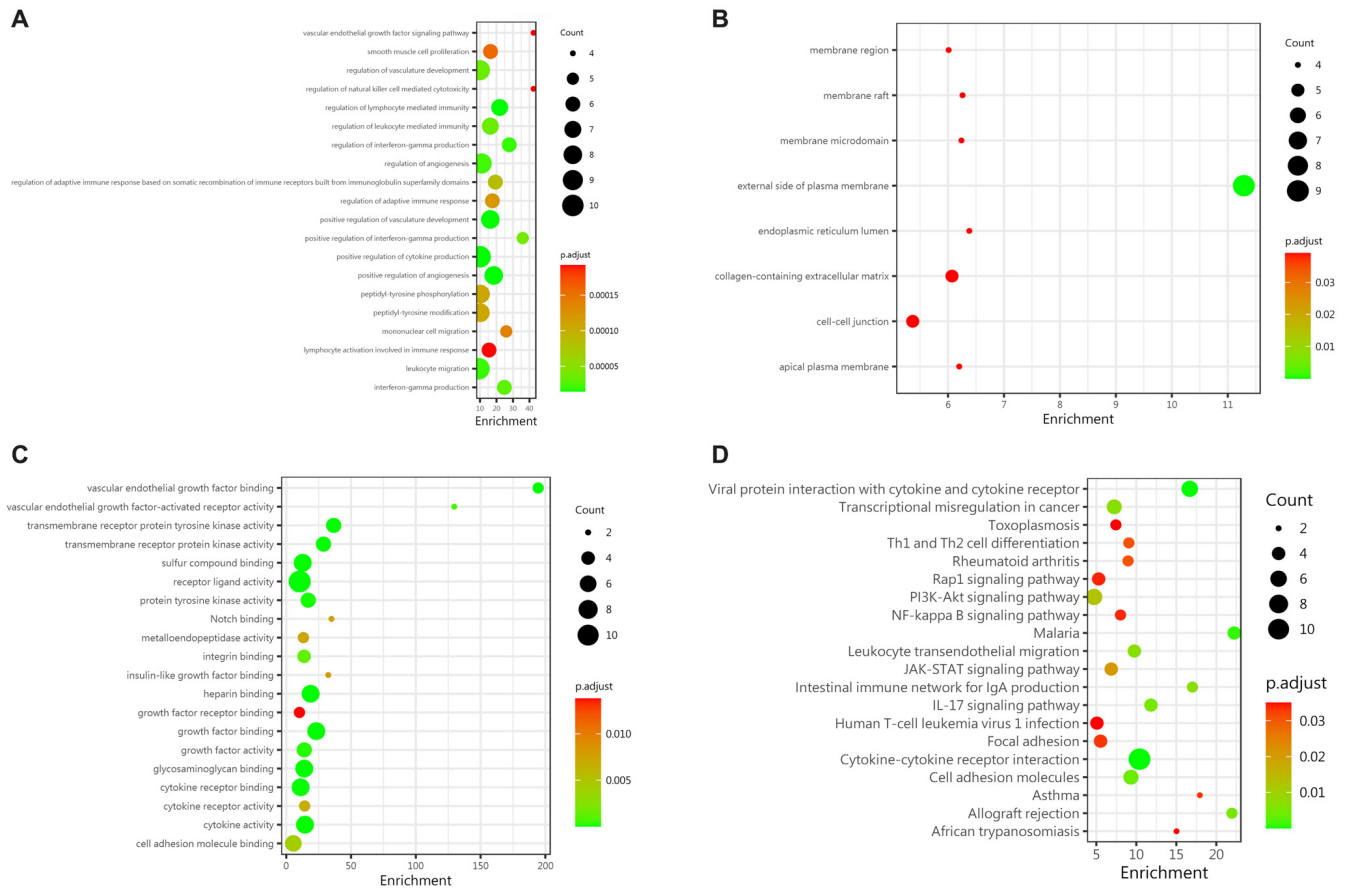
**Biological analysis of differential proteins between G2 and G3.** (A) BPs revealed that the differences between the two groups were primarily related to the regulation of angiogenesis and development, lymphocyte immune regulation, positive regulation of cytokines, leukocyte migration, interferon-γ production and regulation, and tyrosine phosphorylation and modification; (B) In terms of CC, the differences were mainly found in the outer side of the plasma membrane, the ECM containing collagen, intercellular junctions, and the endoplasmic reticulum lumen; (C) Differences in MFs between the two groups were mainly focused on receptor–ligand activity, growth factor binding, heparin binding, transmembrane receptor protein tyrosine kinase activity, vascular endothelial growth factor binding, and cytokine activity; (D) KEGG pathway enrichment analysis identified the most significant pathways, including cytokine–cytokine receptor interaction, viral protein interaction with cytokine and cytokine receptor, malaria, cell adhesion molecules, and PI3K-Akt signaling pathways. BPs: Biological processes; CC: Cellular composition; MFs: Molecular functions; ECM: Extracellular matrix.

Both groups of children developed IgAV, but only a subset progressed to IgAVN. Understanding the biological significance of differentially expressed proteins between these two groups can help reveal the pathogenesis of IgAVN. GO and KEGG analyses identified significant enrichment in 478 GO terms and 22 pathways (adjusted *P* value < 0.05, FDR<0.05). Biological analysis revealed that differences between the two groups were mainly concentrated in angiogenesis regulation, development, lymphocyte immune regulation, positive regulation of cytokines, leukocyte migration, interferon gamma production and regulation, and tyrosine acid phosphorylation and modification (Figure 9A). In terms of CC, the differences were primarily located on the outer side of the plasma membrane, in the collagen-containing ECM, intercellular junctions, and the lumen of the endoplasmic reticulum (Figure 9B). MF differences were mainly concentrated in receptor-ligand activity, growth factor binding, heparin binding, transmembrane receptor protein tyrosine kinase activity, vascular endothelial growth factor binding, and cytokine activity (Figure 9C). KEGG enrichment analysis showed that the differential proteins between the IgAVN group and the IgAV group were most significantly enriched in pathways such as cytokine–cytokine receptor interaction, viral protein interaction with cytokines, malaria, cell adhesion molecules, and PI3K-Akt (Figure 9D).

### Renal tissue validation of differential proteins

**Figure 10.**
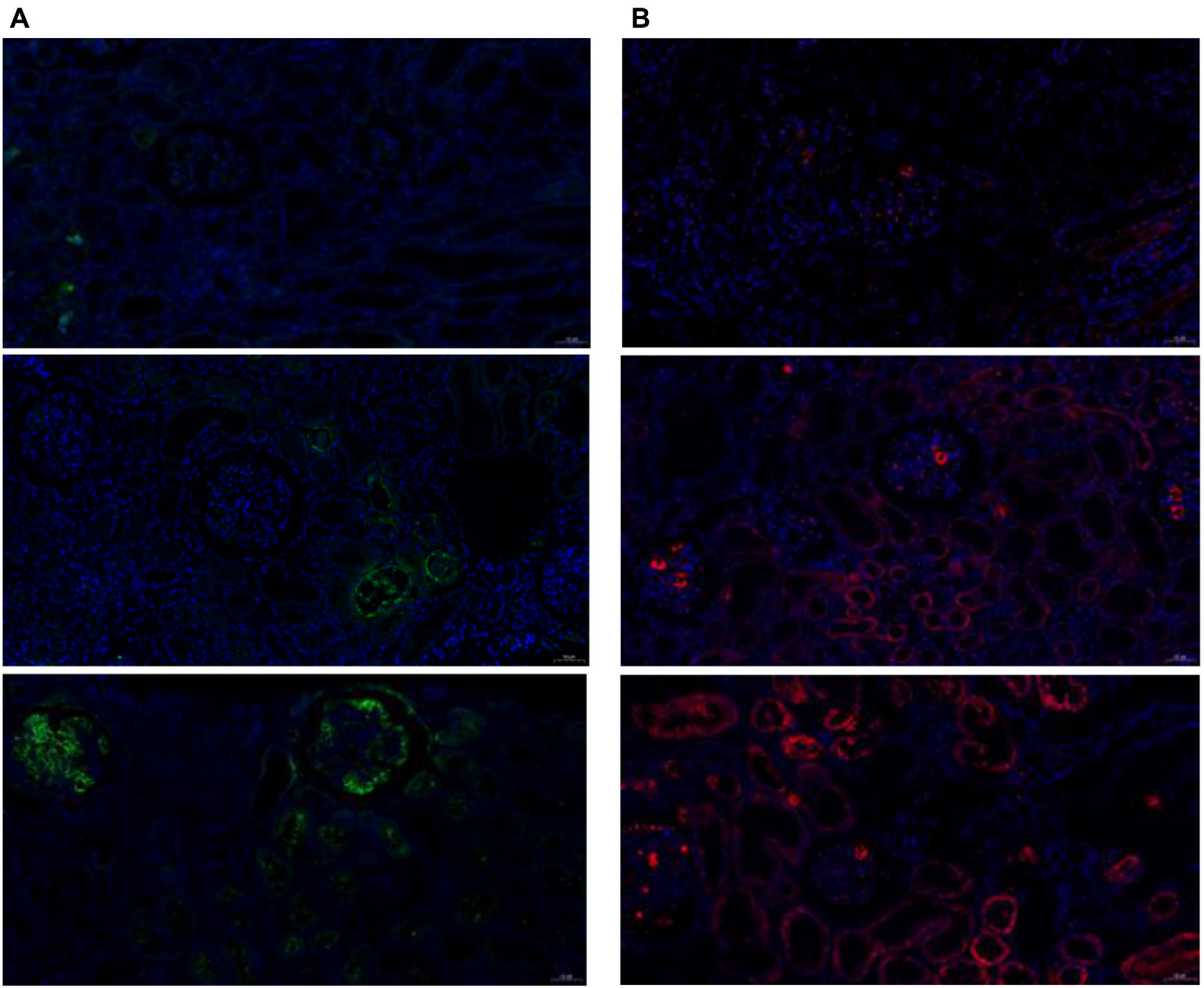
**Immunofluorescence expression of cytokines in kidney tissue (pathological grades from mild to severe, 200× magnification).** (A) Green indicates the expression of ADAM12; (B) Red indicates the expression of TIM-3.

**Figure 11.**
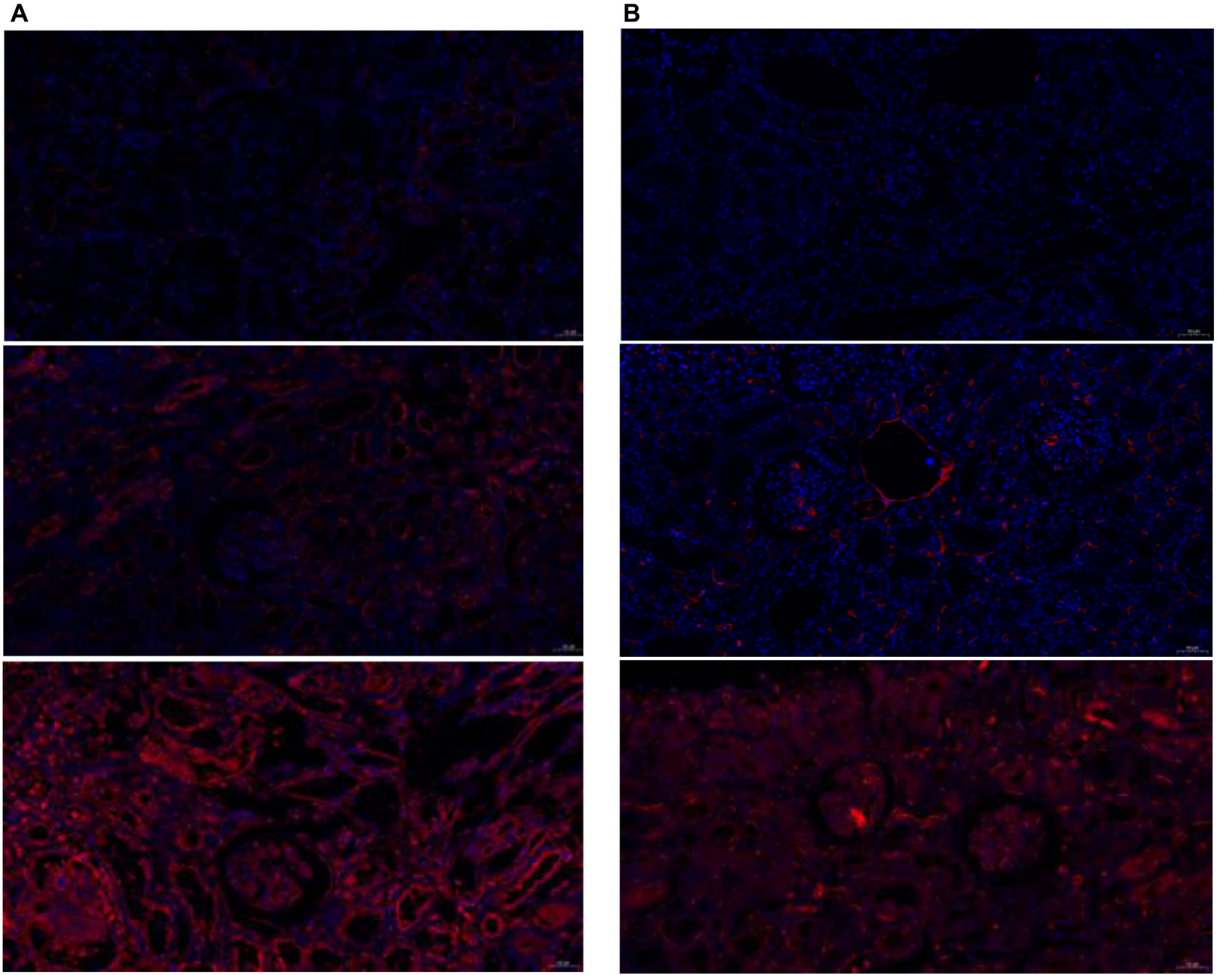
**Immunofluorescence expression of cytokines in kidney tissue (pathological grades from mild to severe, 200× magnification).** (A) Red indicates the expression of IL-12p40; (B) Red indicates the expression of CEACAM-5.

To verify the expression of differential proteins in renal tissue and their relationship with severity and prognosis, we performed immunofluorescence staining of VEGF R3, ADAM12, TIM-3, IL-12p40, and CEACAM-5 in renal puncture tissues, which were the most significantly different proteins between IgAVN and IgAV. The five proteins were not expressed in control renal tissues. However, ADAM12, TIM-3, IL-12P40, and CEACAM-5 were all expressed in IgAVN renal tissue (Figures 10 and 11), with VEGF R3 not expressed.

We classified pathological grades into the mild damage group (ISKDC grade II, *n* ═ 6) and the moderate/severe damage group (ISKDC grade III-VI, *n* ═ 12). The Mantel–Haenszel chi-square test showed a linear relationship between pathological grade and ADAM12/CEACAM-5 expression in children with IgAVN (*P* < 0.05). Pearson correlation results showed that the pathological grade of IgAVN increased with higher ADAM12/CEACAM-5 expression (*P* < 0.05). Furthermore, the results indicated that pathological grade was not related to TIM-3 (*P* > 0.05). Due to non-differentiated distribution, statistical analysis of IL-12p40 was not possible (Table 5).

To further investigate whether the factors with the most significant differences between IgAVN and IgAV are related to the prognosis of IgAVN, we divided the prognosis into the complete remission group (*n* ═ 8) and the persistent abnormal group (*n* ═ 12). The results of the Mantel–Haenszel chi-square test showed that there may be a linear relationship between the prognosis of IgAVN children and CEACAM-5 (χ2 ═ 3.291, *P* ═ 0.07); the Pearson correlation did not suggest a significant statistical difference (*R* ═ 0.440, *P* ═ 0.068), which may be related to the sample size; the analysis showed that ADAM12, TIM-3, and IL-12p40 had no linear relationship with prognosis (Table 6).

## Discussion

In this study, a quantitative planar protein microarray was performed on 440 different proteins to investigate the serum cytokine profile of IgAVN patients, aiming to identify and evaluate potential biomarkers for predicting the risk of future renal involvement at disease onset. Among IgAV patients and healthy controls, 45 proteins were upregulated in the IgAV group, such as CA9, Epo R, IL-1 F9, IL-17C, MMP-3, Angiotensinogen, CD58, and IL-20, while 36 proteins were downregulated, including Cadherin-13, G-CSF R, VEGF R2, TIM-3, IL-1 RII, and CF XIV. Compared to healthy controls, 44 proteins were upregulated and five were downregulated in the IgAVN group. Finally, in the comparison between the IgAV and IgAVN groups, 13 proteins were upregulated in the IgAVN group, including VEGF R3, ADAM12, TIM-3, CEACAM-5, and IL-12p40, while 28 proteins were downregulated, including Resistin, IL-17E, LRP-6, MCP-3, Follistatin-like 1, ICAM-3, TRAIL R3, Tie-1, and Thrombospondin-2.

Our results indicated that the pathways and molecular biological functions of differential factor enrichment are similar when IgAV and IgAVN are compared to the control group alone. The enrichment mainly involved cell migration, chemotaxis, positive regulation of cytokine expression, and responses to various stimuli, indicating that these two diseases are indeed part of the same disease spectrum and are both immune dysregulation disorders. Consistent with other studies, we found that the PI3K/Akt pathway was enriched in all differential factors across the three groups, which may be related to the progression of IgAV and IgAVN, as seen in previous studies [17–19]. The PI3K/Akt signaling pathway is involved in a variety of cellular functions, including cell survival, growth, and protein synthesis [20–22]. Several studies have shown that the PI3K/Akt signaling pathway is associated with renal mesangial cell proliferation [23], renal ischemia/reperfusion injury [24], kidney fibrosis [25], and renal lipotoxicity [26].

However, when comparing the differences between the IgAV and IgAVN groups, we observed significant changes in biological functions and pathways. The differences in IgAV were primarily related to angiogenesis regulation, development, and lymphocyte immunity, whereas IgAVN was more associated with malaria and cell adhesion molecules. Fang et al. [17] found that the cell adhesion pathway was related to IgAVN pathogenesis through urinary proteomics. Cell adhesion is essential for maintaining the mechanical integrity of podocytes, a key component of the glomerular filtration barrier [27]. In addition, we found that vascular-related cytokines were significantly increased in IgAVN, but no specific studies have been conducted on this.

ADAM12 belongs to the ADAM family (disintegrin and metalloproteinase), which is involved in extracellular metalloproteinase activity, cell adhesion, and intracellular signal transduction. As key ectodomain-shedding proteases, ADAMs release various cell surface proteins, including growth factors, cytokines, cell adhesion molecules, and receptors. They can cleave and remodel components of the ECM and are critical regulators of cell–cell and cell–matrix interactions [28]. ADAM12 may regulate cell–cell and cell–matrix contacts through interactions with cell surface receptors (such as integrins and syndecans), which may influence the actin cytoskeleton [29]. ADAM12 expression has been associated with tumor progression and is considered a potential biomarker for breast cancer [30]. Ramdas et al. [31] found that ADAM12 and ADAM19 were involved in renal fibrosis and were regulated by canonical miR-29 and TGF-β. ADAMs and the miR-29 family were therapeutic targets for renal fibrosis. In our study, ADAM12 was positively correlated with the pathological grade of IgAVN, suggesting that it may be a potential biomarker for predicting disease severity. Its role in IgAVN pathogenesis warrants further investigation.

The T cell immunoglobulin and mucin domain-3 (TIM-3) is a unique inhibitory co-receptor. Its expression is predominantly limited to CD4+ and CD8+ T cells that produce interferon γ (IFN-γ). TIM-3 plays a critical role in maintaining the balance between physiological immune responses and maladaptive reactions [32]. Yang et al. [33] found that TIM-3 is pivotal in nephritis and represents a potential therapeutic target for renal injury in diabetic nephropathy. However, in our study, TIM-3 showed no impact on the pathology or prognosis of IgAVN, suggesting further research is necessary.

**Table 5:** Immunofluorescence staining of tissue sections with different proteins at different pathological levels

**ProteinsID**	**Grades**	**Mild damage group (*n* ═ 6)**	**Moderate/severe damage group (*n* ═ 12)**	***χ^2^*** **(*P*)**	*R* **(*P*)**
ADAM12	Negative	0	0	6.800 (0.009)	0.632 (0.005)
	+	0	0		
	++	6	4		
	+++	0	8		
TIM-3	Negative	0	0	2.366 (0.124)	0.373 (0.127)
	+	3	3		
	++	3	5		
	+++	0	4		
IL-12p40	Negative	0	0	ns	
	+	0	0		
	++	0	0		
	+++	6	12		
CEACAM-5	Negative	0	0	6.800 (0.009)	0.632 (0.005)
	+	3	0		
	++	3	8		
	+++	0	4		

**Table 6:** Immunofluorescence staining of tissue sections with different prognosis of different cytokines

**ProteinsID**	**Grades**	**Complete remission group (*n* ═ 8)**	**Persistent abnormal group** **(*n* ═ 10)**	***χ^2^*** **(*P*)**	***R*** **(*P*)**
ADAM12	Negative	0	0	0.266 (0.606)	0.125 (0.621)
	+	0	0		
	++	5	5		
	+++	3	5		
TIM-3	Negative	0	0	0.483 (0.487)	0.169 (0.504)
	+	3	3		
	++	4	4		
	+++	1	3		
IL-12p40	Negative	0	0	ns	
	+	0	0		
	++	0	0		
	+++	6	12		
CEACAM-5	Negative	0	0	3.291 (0.070)	0.440 (0.068)
	+	2	1		
	++	6	5		
	+++	0	4		

The carcinoembryonic antigen (CEA, also known as CD66e and CEACAM-5) is a cell surface glycoprotein commonly overexpressed and released by many solid tumors, playing an autocrine role in cancer cell survival and differentiation. Soluble CEA, released by tumor cells and found in the circulatory system, can induce proangiogenic behavior in endothelial cells, including adhesion, spreading, proliferation, and migration *in vitro*, as well as tumor microvascular formation *in vivo* [34]. Currently, no studies have explored the role of CEACAM-5 in the kidneys. Our results demonstrated a positive correlation between CEACAM-5 expression and pathological grade—the stronger the expression, the more severe the pathological grade. Additionally, CEACAM-5 may be associated with prognosis. However, the exact mechanism remains unclear, necessitating further investigation. CEACAM-5 has the potential to become a biomarker for disease severity and prognosis. In the future, we plan to expand the sample size for verification and conduct mechanistic studies.

The underlying mechanisms of IgAV remain unclear, though recent studies suggest that FMF may play a role in IgAV development [35]. Our study indicates that the high-throughput cytokine chip is an ideal method for detecting differential serum cytokines in IgAVN and IgAV, offering new insights into IgAVN progression in children and identifying potential biomarkers and mechanisms. We hope these differentially expressed proteins will provide clues to the BPs behind IgAVN onset and development, and that the proteomic profiles may offer guidance for treating and predicting IgAVN prognosis in children. However, there are limitations to our study. The small number of IgAVN patients not receiving hormone treatment and the high cost of the cytokine chip limited this study to serum samples from only 24 patients. As this was a preliminary screening, 18 kidney biopsy specimens were included. In future studies, we aim to collect more serum, urine, and renal biopsy specimens from IgAVN patients to validate potential molecular markers and further investigate the mechanisms of disease onset and progression.

## Conclusion

Our study identified serum cytokine profiles in children with IgAVN and uncovered novel serum cytokines that may serve as potential biomarkers for the condition. We found that the levels of ADAM12 and CEACAM-5 were positively correlated with the severity of IgAVN, as indicated by pathological grading. Notably, CEACAM-5 may also be associated with the prognosis of IgAVN. Consequently, CEACAM-5 could potentially be used as a biomarker to assess disease severity and monitor its progression.
